# Porous Polymers from High Internal Phase Emulsions as Scaffolds for Biological Applications

**DOI:** 10.3390/polym13111786

**Published:** 2021-05-28

**Authors:** Stanko Kramer, Neil R. Cameron, Peter Krajnc

**Affiliations:** 1 PolyOrgLab, Faculty of Chemistry and Chemical Engineering, University of Maribor, Smetanova ulica 17, 2000 Maribor, Slovenia; stanko.kramer@um.si; 2 Department of Materials Science and Engineering, Monash University, 22 Alliance Lane, Clayton, VIC 3800, Australia

**Keywords:** polyHIPE, tissue engineering, cell culturing, emulsion templating, porous polymers, biodegradable polymers

## Abstract

High internal phase emulsions (HIPEs), with densely packed droplets of internal phase and monomers dispersed in the continuous phase, are now an established medium for porous polymer preparation (polyHIPEs). The ability to influence the pore size and interconnectivity, together with the process scalability and a wide spectrum of possible chemistries are important advantages of polyHIPEs. In this review, the focus on the biomedical applications of polyHIPEs is emphasised, in particular the applications of polyHIPEs as scaffolds/supports for biological cell growth, proliferation and tissue (re)generation. An overview of the polyHIPE preparation methodology is given and possibilities of morphology tuning are outlined. In the continuation, polyHIPEs with different chemistries and their interaction with biological systems are described. A further focus is given to combined techniques and advanced applications.

## 1. Introduction

Human life expectancy has been increasing gradually over the years [1]. This increase can be associated with the advancement of medicine, public health availability and healthier lifestyles [2,3]. However, the increase in life expectancy comes with many issues related to ageing, including tissue and organ failure [4]. Currently tissue repair is generally conducted by transplanting the tissue from a healthy donor (allograft) or the patient’s own body (autograft). However, these procedures are flawed due to the lack of donor tissues, donor sites, potential infections and low allograft survival rates, to name a few [5,6]. A growing field that can avoid these limitations is tissue engineering. Tissue engineering uses natural, synthetic or semi-synthetic tissues to replace damaged tissues [7]. Generally, tissue engineering combines scaffolds, cells and growth factors to form appropriate environments for the formation of new tissues [8]. The scaffolds used in tissue engineering need to fulfil certain requirements to be suitable for such applications. They need to have an appropriate biocompatibility (cells must adhere, function normally, migrate onto the surface and through the scaffold), biodegradability (the implanted scaffold needs to degrade in order for the cells to replace it), mechanical properties (comparable to the site of implantation) and scaffold architecture (interconnected pore structure and high porosity) [9]. One promising type of scaffold is porous polymers obtained through emulsion templating. These scaffolds fulfil the architectural requirements that are vital for scaffolds used in tissue engineering. Additionally, they can be produced from a plethora of different reagents which enable the synthesis of materials with appropriate biocompatibility, biodegradability and mechanical properties.

**Figure 1:**
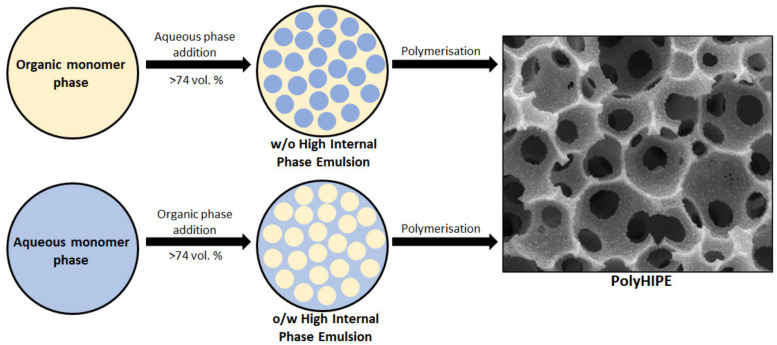
Schematic representation of the formation of a polyHIPE.

**Figure 2:**
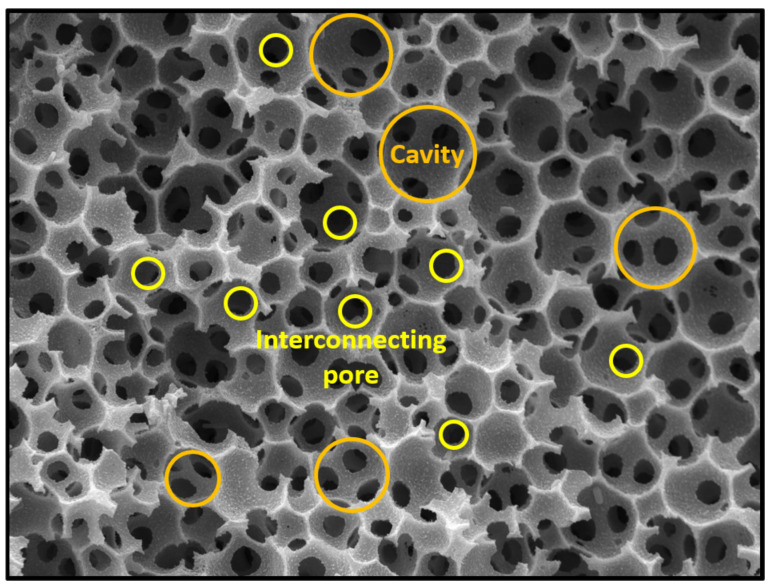
Typical polyHIPE morphology consisting of cavities and interconnecting pores.

Porous polymers can be produced using several methods like direct templating, block copolymer self-assembly, direct synthesis, breath figures and high internal phase emulsion (HIPE) polymerisation, to name a few [10]. The main advantage of HIPE polymerisation is their ability to produce a highly porous and a three-dimensional highly interconnected scaffold, which facilitates cell ingrowth [11]. HIPEs are formed by mixing two immiscible liquids where one is usually the oil (organic) phase while the other is the water (aqueous) phase [12]. These phases can form a water-in-oil (w/o) or oil-in-water (o/w) emulsion. Essentially, one of the phases represents the internal (dispersed) phase, which has a volume fraction of at least 74.05% (maximum space that can be filled by uniformly sized spheres) or 64% (for random close packing), whereas the other represents the external (continuous phase) [13,14,15,16,17]. The formation of either a w/o or an o/w emulsion is dictated by the surfactant. If the surfactant is predominantly soluble in the oil phase a w/o emulsion is formed and if it is predominantly soluble in the water phase an o/w emulsion is formed [18]. After the formation of a stable HIPE, the continuous phase is polymerised and a polyHIPE is formed (Figure 1). PolyHIPEs have a hierarchically porous structure consisting of primary pores (cavities) and secondary, interconnecting pores (Figure 2). Both the chemical variety and the high porosity variety enable the formation of materials for specific applications, for instance, water clean-up [19], absorption and adsorption [20,21], separation processes [22,23], controlled release matrices [24] and tissue engineering [25]. Another aspect that makes polyHIPEs attractive is the available polymerisation techniques, such as atom transfer radical polymerisation (ATRP) [26], free-radical polymerisation (FRP) [27], reversible-addition-fragmentation chain-transfer (RAFT) [28], ring opening metathesis polymerisation (ROMP) [29] and click polymerisation [30], to name a few. In addition to building blocks, another property that can be freely controlled is the morphology of the prepared polyHIPEs. This can be done by increasing the internal phase volume, which increases the interconnectivity and the pore diameter [31]. The addition of different additives (solvents) also leads to an increase of both the cavity and interconnect diameters, due to the partial destabilisation of the emulsion [32]. Lastly, the pore size can also be adjusted through temperature control (increasing the temperature destabilises the emulsion and increases the cavity diameter) and stirring speed [33]. Besides the adjustability possible before the polymerisation, polyHIPEs can also be modified after the polymerisation, which is often necessary, as polyHIPEs tend to have a hydrophobic surface (prevents cell attachment). The surfaces can be made more hydrophilic by functionalising the surface with hydrophilic molecules or through plasma treatment [34,35,36].

All of these aspects (chemical versatility, tunability of the morphology, post-polymerisation modification) make polyHIPEs an interesting material for several types of research, including the development of artificial tissues. Due to that, several studies have been conducted on the applicative possibilities of polyHIPEs in tissue engineering.

## 2. Cell Culturing and Tissue Generation on PolyHIPEs

### 2.1. Styrene-Based PolyHIPEs

Styrene-based polyHIPEs are the most studied type of porous polymers synthesised from high internal phase emulsion templating. PolyHIPEs based on styrene (STY) have been shown to significantly increase the cell growth of human liver cancer cells (HepG2) when compared to 2-D (two-dimensional) tissue culture plastic. The cells were shown to have high levels of cell viability, excellent cellular morphology (more homogeneous than the cells grown on 2-D, various mitochondria, presence of peroxisomal clusters) and greater functions than the cells grown on 2-D plastic. This demonstrates the great potential of polyHIPEs for 3-D (three-dimensional) cell culture [37]. The research of cell growth on such scaffolds was further investigated by preparing styrene-co-divinylbenzene (STY-co-DVB) scaffolds with a nominal porosity of 80%. Two-dimensional standard support was compared to the synthesised scaffolds by using HepG2 cells. The cells were successfully cultured for up to 21 d on both substrates, however, the cells formed flat structures and were heterogeneous on the 2-D scaffold. Additionally, they appeared unhealthy and started to disintegrate at 14–21 d. In comparison, the cells grown on the polyHIPEs spread on the surface and in the inner parts of the materials and appeared more homogeneous. The cells grown on the 3-D scaffolds also had a significantly greater number of microvilli compared to the cells cultured on the 2-D surfaces. To further demonstrate the difference of 3-D and 2-D scaffolds, the cytotoxic tolerance of the scaffolds was assessed by using methotrexate (MTX). Low concentration of MTX had significant effect on the metabolic activity of HepG2 cells grown on the 2-D scaffolds, whereas there were no significant effects on the cells cultured on the 3-D scaffold. To affect the cells in the polyHIPEs, higher concentrations of MTX were required which implies that the cells grown in 3-D scaffolds are more tolerant to the cytotoxins. It was also shown that at the highest concentration of MTX, the cells grown on 2-D materials had undergone advanced stage of cell death, whereas in the polyHIPEs, the nuclear membrane had irregular morphologies and abnormal features on the mitochondria. This demonstrated that cells grown on 3-D scaffolds undergo the same changes as on 2-D scaffolds, but at considerably higher concentrations of MTX which means that they are more tolerant to the toxins. The studies done on the polyHIPEs are also more likely to closer mimic real physiological environments [38]. The main reason polyHIPEs are able to mimic real physiological environments closer is their porous interconnected morphology. Given this inherent property of polyHIPEs, the ability to control the size and interconnectivity of the prepared scaffolds is an essential research topic. Due to this, Bokhari et al. [39] investigated the method of internal phase addition. The scaffolds researched were based on styrene (STY), 2-ethylhexly acrylate (EHA) and divinylbenzene (DVB) with a porosity of 90%. This study showed that by adding the aqueous (internal) phase in a controlled manner it is possible to produce pores with a narrower size distribution. Additionally, the reproduction of the emulsions becomes feasible. The obtained polyHIPE had an average pore diameter of 90 μm while the diameter of the interconnecting pores was approximately 20 μm. This polyHIPE enabled successful cell attachment and growth. The bone osteosarcoma cells (MG63) grew throughout the cavities and linked together, fully colonising the entire structure of the scaffolds after a certain period. The interaction of the cells with neighbouring cells creates a cellular structure comparable to the native structure. The polyHIPE was also compared to a 2-D scaffold and was shown to be superior in all regards. This was especially visible in the levels of alkaline phosphatase which were greater in the 3-D scaffold [39]. The advantages of polyHIPEs over 2-D scaffolds were also demonstrated by evaluating the cytotoxicity of cigarette smoke. It was shown that the prepared STY-co-DVB polyHIPE with a porosity of 95.3%, a pore diameter of 12 μm and interconnect diameter of 2.61 μm enabled the successful proliferation of adenocarcinomic human alveolar basal epithelial cells (A549). This scaffold was also used in cytotoxicity evaluation tests of cigarette smoke. The cells cultured on the polyHIPEs had considerably higher cell viabilities than the ones cultured on 2-D scaffolds after the total particulate matter (TPM) exposure (71.4% vs. 46.8% when exposed to 120 μg/mL TPM for 24 h). The lower cytotoxicity of smoke in the 3-D system is most likely due to the formation of a 3-D system similar to in vivo, which would affect the transport of nutrients and waste, and TPM permeability resulting in a higher cell viability. This demonstrates the ability of polyHIPE scaffolds to be used in cytotoxicity evaluation studies [40].

As polystyrene-based scaffolds are inherently hydrophobic, it is often necessary to modify them appropriately to facilitate cell attachment. One way to do that is through air-plasma treatment which increases the hydrophilicity of the scaffold and therefore, cell attachment. The use of this method has been demonstrated on polyHIPEs based on STY and ethylene glycol dimethacrylate (EGDMA). The water contact angle dropped from 128.7 to 10.3° after air plasma treatment (increased hydrophilicity). The enhanced properties of the scaffold were observed in cytotoxicity tests, cell attachment and cell proliferation. The cytotoxicity of the treated material was 98%, whereas the untreated material had a cytotoxicity of 91%. This test demonstrated that neither of the materials were cytotoxic. A greater difference was observed in cell attachment and cell proliferation studies. The air-plasma treated scaffold was shown to have significantly higher cell attachment and cell proliferation when compared to the untreated scaffold. This is most likely due to the increased hydrophilicity [41]. PolyHIPEs can also be modified by functionalising their surfaces with different functionalities, for example, acrylic acid (AA). Scaffolds based on STY, DVB and EHA were functionalised with AA which was added to the internal aqueous phase to functionalise the surface. The obtained polyHIPE had a porosity of 89% and an open porous morphology with an average pore diameter of 19 µm. The scaffold was shown to enable the cell attachment of hepatocytes which was comparable to the commercially available Alvetex. Due to the functionalisation of the surface, further surface modifications and bioconjugations are feasible, which increases the material’s versatility [42]. In a different study, the styrene-based scaffold was functionalised with pentafluorophenyl acrylate (PFPA) to enable further functionalisation with galactose, as galactose binds to hepatocytes to promote cell adhesion and cell function via the asialoglycoprotein receptor (ASGRP). The prepared polyHIPE had a porosity of 92%; and a pore and interconnect diameter of 33 and 10 µm, respectively. These scaffolds were suitable for hepatocyte cell culture. After the attachment of 2′-aminoethyl-β-D-galactopyranoside to the PFPA scaffold, the cell tests showed a significant increase in cellular albumin synthesis, which indicates the accessibility of the carbohydrates to interact with the cells [43]. Another type of molecule which enhances the properties of scaffolds is hydroxyapatite (HA). Hydroxyapatite has great biocompatibility, high bioactivity, high osteoconductivity and is non-cytotoxic [44]. These properties make it an attractive molecule to functionalise polyHIPEs with. Due to this, Akay et al. [45] prepared three types of HA-functionalised polystyrene-based scaffolds with an internal phase volume of 95%; a pore size of 100, 60 and 40 µm; and an interconnect size of 30, 20 and 15 µm, respectively. The polyHIPEs were shown to significantly increase the amount of osteoblast cells at both 24 h and 35 d of culture when compared to tissue culture plastic (TCP). However, no significant differences were observed between the pore sizes. The cells grew mainly on the surface of the polyHIPE and only a few cells penetrated into the polymer up to a depth of 30 µm (day 14). However, at 35 d the maximum depth of penetration was 1.4 mm on the HA modified polymers. The depth of penetration did not differ greatly between the scaffolds with different pore sizes. The main difference was in the rate of penetration which was noticeably quicker in the 100 μm polyHIPE. The pore size did not seem to affect cell growth significantly, unlike HA addition, which was shown to increase penetration and proliferation of the osteoblasts [45]. As HA was demonstrated to increase cell functions it was also used by Sapsrihong’s group [46] when preparing a poly(styrene/ethylene glycol dimethacrylate) scaffold. The polyHIPEs had an internal phase volume of 90%; an open porous structure with a pore diameter between 30 and 40 µm; a compressive modulus of 18 MPa and a compressive strength of 0.9 MPa. To further enhance the material’s adhesive properties and interaction with living tissue it was coated with a nanolayer by using the layer-by-layer polyelectrolyte multilayer (PEM) technique. The primary layer was made up of seven layers of poly(diallyldimethylammonium chloride) (PDADMAC), whereas for the secondary coating, poly(sodium 4-styrene sulfonate) (PSS), alginic acid (ALG) and gelatine (GEL) were used. The PEM nanolayer decreased the contact angle from 122 for unmodified to 55, 72 and 0° for PSS, ALG and GEL modified samples, respectively. None of the prepared polyHIPEs were shown to leach any toxic products, as the amount of living fibroblasts (cell line L929) from subcutaneous connective tissues of mice was 93% for the PSS modified polyHIPEs (highest) and 77% for the GEL modified polyHIPEs (lowest) when compared to the control scaffold (TCPs). The PSS modified poly(S-EGDMA)HIPE showed the highest efficiency of cell attachment and cell proliferation of all the modified scaffolds. When compared to the unmodified scaffold, it had a 60% higher cell amount. This makes the PSS-modified scaffold a good candidate for various biological applications [46]. Another way to functionalise styrene-based polyHIPEs is by using amphiphilic copolymers which functionalise the surface and also act as a surfactant. Great examples of such copolymers are polystyrene-*b*-poly (ethylene oxide) (PS-PEO) and polystyrene-*b*-poly (acrylic acid) (PS-PAA), which were used to prepare scaffolds with an internal phase volume of approximately 80% and a pore diameter ranging from 151 (0% PEO) to 64 µm (100% PEO). PS-PAA and PS-PEO were used to functionalise the scaffold with cell adhesive (PAA) and cell inert (PEO) domains to form a heterogeneous surface which would mimic the heterogeneity of the extra cellular matrix (ECM). To evaluate this material for cell-related properties, human embryonic stem cell-derived mesenchymal progenitor cells (hES-MPs) were used. The hES-MPs were shown to adhere in a composition dependent manner, especially in regard to the topologies which mimic the heterogeneity of adhesive sites in native ECM. The lowest adherence and proliferation were on the PEO100 and PAA100 foams with the PEO75 foams having the highest number of cells after 7 d [47]. This study was further upgraded by using the amphiphilic copolymers poly (1,4 butadiene) poly(ethylene oxide) (PBD-PEO) and PS-PAA to prepare scaffolds with an internal phase volume of 90%, and either an open or a closed porous structure with a pore diameter ranging from 40 to 80 µm. It was shown that culturing hES-MPs for a period of 7 d favoured open porous scaffolds, as they had higher cell viability compared to the closed porous scaffolds. Additionally, the mixed copolymer compositions (50% PEO and 75% PEO) had higher cell viability than the single copolymer compositions. The cells cultured on the open porous foams adhered to the surface and also spread into the inner parts due to the open porosity, whereas the cells on the closed porous material only adhered to the surface. Another difference between the open and closed porous scaffolds was the duration of cell proliferation which was up to 28 d on the open porous scaffolds and only up to 7 d on the closed porous scaffolds. It was also demonstrated that the different copolymer compositions in open porous foams have the potential to differentiate hES-MP cells, mainly towards osteogenic differentiation [48].

Besides chemical modifications, polymers can also be modified through coatings. One of the molecules that can be used is HA, which was already been shown to enhance cell functions [49]. Some other reagents that can be used to coat the surface and enhance cell adherence are poly-D-lysine and laminin. Poly-D-lysine is a chemically synthesised ECM and laminin is a protein present in the ECM. Both are used to facilitate cell adhesion [50]. Hayman et al. [51] prepared a styrene-based polyHIPEs with an internal phase volume of approximately 90% and a cavity diameter ranging from 50 to 100 µm. The prepared polyHIPEs were coated with poly-D-lysine, laminin or both. To test the material’s biocompatibility, human pluripotent embryonal carcinoma stem cells (TERA2.cl.SP12) were used. A large number of neurons adhered onto the poly-D-lysine and poly-D-lysine plus laminin scaffold, however, the amount of viable cells on the laminin only coated scaffold was approximately 40% lower. These results indicate that poly-D-lysine is the one responsible for the successful attachment of neurons onto the polyHIPE scaffold. It was also shown that by using the combination of both poly-D-lysine and laminin significantly longer neurite extensions are produced when compared to the poly-D-lysine only coating.

As styrene-based polymers are inherently non-biodegradable their use in tissue engineering applications is limited. Therefore, combining them with biodegradable monomers is an interesting way to enhance their properties. Busby et al. [52] prepared styrene-based polymers which had a 20 wt. % content of either poly(ε-caprolactone) (PCL) or poly (lactic acid) (PLA). These materials enabled cell adhesion, proliferation and differentiation of three different types of tissue (whole chicken embryo explants, rat skin explants, individual human fibroblasts). Another styrene-based polyHIPE prepared with PCL and an internal phase volume of 90% was shown to enable the growth of human fibroblasts [53]. Overall, this shows the ability of PCL containing scaffolds to enable the cell growth of different types of cells.

Another type of macromolecules which STY can be combined with are peptides. Bokhari et al. [54] prepared a peptide hydrogel-polyHIPE hybrid by combining a styrene-based polyHIPE and a peptide hydrogel (RAD16-I) to enhance osteoblast growth. The hybrid scaffold supported osteoblastic growth of primary rat osteoblasts. The ECM deposited on both the scaffold surface and within the scaffold. The incorporation of RAD16-I also affected the appearance of the cells, which was more fibroblastic, compared to the polyHIPE only scaffold. This indicates good cell adhesion. The cells also grew into the scaffold to a maximum depth of 3 mm. It was shown that the incorporation of RAD16-I within a polyHIPE significantly increased the scaffold’s osteoblast activity and bone formation. Besides the traditional formation of chemically bonded networks, an intriguing way to prepare polyHIPEs is by using semi-interpenetrating polymer networks (semi-IPN). Lumelsky et al. [55] combined PCL with polystyrene to form such a polymer. These scaffolds were shown to have considerably larger cavities (up to 285 µm) when compared to the polystyrene polyHIPEs which had a pore diameter of up to 30 µm. These semi-IPN polymers were shown to enable the attachment of mouse skeletal C2 cells. The cells managed to form a monolayer, spontaneously differentiated and formed myotubes.

Despite the good properties demonstrated by styrene-based scaffolds, their use in tissue engineering is somewhat limited, due to their inherent non-degradable properties. However, as they have been well studied, a commercially available polystyrene-based polyHIPE (Alvetex) has been produced. This scaffold is widely used for routine 3-D cell culture which is also the most suitable field of application for styrene-based scaffolds. A summary of the described polyHIPEs is shown in Table 1.

### 2.2. Acrylate-Based PolyHIPEs

Unlike styrene-based polyHIPEs, acrylate-based ones are degradable to a certain extent and have a larger selection of monomers that can be used in the polymerisation procedures. Examples of such monomers are ethylene glycol dimethacrylate (EGDMA), propylene fumarate dimethacrylate (PFDMA), trimethylolpropane triacrylate (TMPTA), EHA, butanediol dimethacrylate (BDMA), glycerol monomethacrylate (GMMA) and hydroxyethylmethacrylate (HEMA), to name a few [56,57,58]. For example, EGDMA and PFDMA have been used for the culture of human mesenchymal stem cells (hMSCs). Both have been shown to have high cell viabilities of the seeded hMSCs (90% in the case of PFDMA) [56]. A copolymer made up of EGDMA, PFDMA and BDMA was also shown to have high cell viability for hMSCs [57]. Another copolymer that can be used for biological applications is comprised of EHA and TMPTA. This scaffold was shown to facilitate the cell growth of the murine long bone osteocyte cell line (MLO-A5) on both the surface and the inner parts [59]. Another property that makes acrylates more suitable for biological applications is the ability to synthesise hydrogels. Hydrogels are inherently hydrophilic which means that further modifications to increase the hydrophilicity are not required. To prepare acrylate-based hydrogels, Nalawade et al. [58] synthesised a polyHIPE based on GMMA, HEMA and GDMA. The polyHIPE had porosities ranging from 92.3% to 96.6%, pore diameters ranging from 20 to 30 µm and interconnect diameters ranging from 3.7 to 7.7 µm. As the prepared material is hydrophilic it enables the absorption of water which facilitates the transport of nutrients and waste. The prepared polyHIPE had swelling ratios ranging from 8.1 to 13.6, increasing with the porosity. As degradation is another important property it was also evaluated. The degradation in 0.007 M NaOH after 10 d was approximately 26%. All these favourable properties enabled a high cell viability of greater than 97% for the mouse fibroblasts (NIH/3T3 cell line). Additionally, the observed cells were shown to be healthy. A different type of hydrophilic arylate was prepared by McGann et al. [60] by using sodium acrylate, calcium acrylate, poly(ethylene glycol) diacrylate (PEGDA) and poly(N-isopropylacrylamide) (PNIPAM), where appropriate. PNIPAM was shown to increase both the pore diameter and interconnect diameter, however, with the addition of PNIPAM, the buffer uptake was reduced. The cytocompatibility of these materials has shown that they are non-toxic for immortal cancer HeLa cells. However, the cells did not spread in great numbers on the surface of the polyHIPE.

Despite the favourable properties of acrylates, further modifying them enhances these properties even more. A commonly used modifying procedure which makes surfaces more hydrophilic is plasma treatment. This method was used by Owen et al. [61] to increase the hydrophilicity of scaffolds based on EHA, isobornyl acrylate (IBOA) and TMPTA. The polymers were treated with either an air plasma clean or air plasma followed by plasma deposited acrylic acid. The scaffolds that were not treated with the plasma were unable to support the attachment of human embryonic-derived mesenchymal progenitor cells (hES-MPs) and therefore, growth. However, both plasma procedures were successful in that regard with no significant differences in metabolic activity of the cells. Another polymer based on the same monomers and treated with plasma was prepared by Paterson et al. [62]. The main difference between their procedure and the aforementioned one was the method of preparation. Paterson et al. [62]. used water-in-oil-in-water multiple emulsions to prepare porous beads. These beads were prepared from a HIPE with an 80% internal phase by using either microfluidics or a controlled stirred-tank reactor (CSTR). The produced beads had a diameter of approximately 300 μm for the beads produced by microfluidics and a diameter of 286 μm for the CSTR produced beads. Another difference was the size distribution of the beads which was considerably narrower in the case of microfluidics. After modifying the produced beads with inductively coupled plasma polymerisation of acrylic acid, hES-MPs were cultured on the polymers. The cells were shown to form large aggregates of proto-tissues with significant increase in cell activity after 11 d of culture. Further tests showed that the cells grew close to the centre point of the spheres (100 μm) after 30 d of growth in osteogenic media. Besides plasma treatment to improve biological activities, the acrylate-based polyHIPEs can also be modified by the addition of different particles. Examples of such particles are calcium phosphate nanoparticles and demineralised bone matrix (DBM) particles. The addition of these particles improved both cell viability and cell proliferation of hMSC cells, while having a negligible effect on the open porous morphology and compressive properties [63]. Lastly, the properties of these acrylates can also be improved by loading substrates onto them. This has been demonstrated on a poly (ethylene glycol) methacrylate (PEGMA) and PEGDA copolymer which was loaded with kaolin. The material was shown to be non-cytotoxic but did not enable the adherence of HFDs [64]. A summary of polyHIPEs based on acrylate is shown in Table 2.

### 2.3. Thiol-Ene-Based PolyHIPEs

PolyHIPEs based on thiol-enes are also a group of degradable polymers. They undergo hydrolytic degradation due to the presence of ester bonds. Additionally, thiol-ene polymerisation has an advantage over other polymerisation due to the formation of a homogeneous polymer network (control over step-growth and chain-growth), high yields and insensitivity to oxygen inhibition [71]. Unlike acrylate-based polymers, thiol-ene-based polymers combine thiols and a plethora of compounds containing double bonds (acrylates, vinyl esters and other alkenes). This ability gives rise to a wide variety of chemistries. Sušec et al. [72] prepared a thiol-ene polyHIPE based on a tetra functional thiol, namely, pentaerythritol tetrakis(3-mercaptopropionate) (PETMP) and divinyl adipate (DVA) with an internal phase volume of 85%. The prepared scaffold had a pore diameter of 13 µm and an interconnect diameter of 2 µm. This open porous scaffold was shown to significantly increase cell multiplication and cell growth of the murine-derived pre-osteoblastic MC3T3-E1 cell line. This type of polyHIPE was further researched by increasing its pore diameter and interconnect diameter to 82 µm and 13 µm, respectively. The scaffold was also shown to be degradable in both phosphate buffer saline (PBS) (45% mass loss after 6 weeks) and in a 0.01 M NaOH solution (full degradation after 4 weeks). The mechanical testing showed that the Young’s modulus of the polyHIPE was 0.15 MPa and 0.18 MPa for the polyHIPE-chondrocyte sample (1 d of culture), which corresponds to 38% and 45% of the human articular cartilage Young’s modulus. This means, that the mechanical properties are adequate to support proliferation and cartilage growth. The chondrocytes formed a multilayered cell film on the polyHIPE surface after 7 d of culture. After 16 d the cells were shown to possess round nuclei on the surface and migrated into the scaffolds to a depth of approximately 300 µm [73]. Caldwell et al. [74] prepared a different type of thiol-ene polymer by polymerising the trifunctional thiol, trimethylolpropane tris(3-mercaptopropionate) (TMPTMP), with dipentaerythritol penta/hexa-acrylate (DPEHA). The polyHIPE had a nominal porosity of 90%, a pore diameter of 108 μm and an interconnect diameter of 18.4 μm. Like the previously mentioned tetrathiols, the trithiols also degraded in 1 M NaOH (full degradation) and 0.1 M NaOH (20% TMPTA, 30% DPEHA). The scaffold was also shown to possess favourable properties in the culture of HaCaT cells as it was shown to be biocompatible. However, unlike in the case of the tetrathiol polyHIPE, the trithiol one had limited cell penetration into the inner parts of the scaffold.

Some other types of acrylates that can be used to prepare thiol-ene polymers from TMPTMP are 1,6-hexanediol diacrylate (HDDA), trimethylolpropane triacrylate (TMPTA) and poly (ethylene glycol) diacrylate (PEGDA). The synthesised materials had a porosity ranging from 77% to 85%, with PEGDA having the highest (85% internal phase vs. 80% internal phase in the others). The average pore diameters of the prepared scaffolds were 30.3, 44.2, 45.3 and 63.2 µm for TMPTA, HDDA, PEGDA and PEGDA_85%, respectively. All of them were capable of absorbing PBS, however, the PEGDA polyHIPEs had an absorption of seven times its own weight, whereas the TMPTA and HDDA polyHIPEs absorbed about 100% of their weight. Additionally, the elastic moduli were also shown to be favourable with PEGDA_85% having its elastic modulus (13 kPa) in the range of the mammalian brain elastic modulus (0.1–24 kPa). As materials for tissue engineering are usually inside a wet environment, testing the properties of these artificial scaffolds under such conditions is also important. That is why this study also evaluated the mechanical properties in PBS at 37 °C to mimic in vivo conditions. Both PEGDA polyHIPEs were shown to have an elastic modulus value in the range of mammalian brain tissue (18.4 kPa and 8.6 kPa). A crucial requirement for tissue engineering applications is also degradability which these materials fulfilled. Lastly, the evaluated scaffolds were successful in the culture of induced pluripotent stem cell (iPSC)-derived human neural progenitors (hNPCs) for a period of 3 d. All of the materials had cell densities comparable to that of the commercially available Alvetex scaffold and enabled the distribution of the HNPCs throughout the entire structure of the scaffolds. The cells were also demonstrated to stain positively for the expression of the protein vimentin [75].

Despite the good properties demonstrated by the previous polyHIPEs, there are several ways to improve them. One way to improve these materials is by functionalising them with different molecules. Richardson et al. [76] functionalised a scaffold with an internal phase volume of 80% and an average pore diameter of 20 to 30 μm based on TMPTMP and TMPTA with N-sulfosuccinimidyl-6-(4′-azido-2′-nitrophenylamino) hexanoate (sulfo SANPAH). This compound enabled further functionalisation with PEG-bis-amine and fibronectin which increased the hydrophilicity of the polymer. The functionalisation with fibronectin resulted in an increase of cell adherence of human embryonic stem cells (HESCs). By day 5 of culture, significant cell infiltration in the sulfo SANPAH/fibronectin scaffold was observed and a 122% increase of cells on the surface compared to the fibronectin scaffold. This indicates that the sulfonate groups affect and also promote cell attachment. With this method cell interaction properties can be enhanced without affecting the physical properties of the polyHIPE (porosity and morphology). Another molecule which successfully modifies this type of polyHIPEs is maleimide. Ratcliffe et al. [77] demonstrated the ability of maleimide to significantly increase cell attachment, proliferation and infiltration of the human embryonic stem cell line WA09 (H9) when compared to the unfunctionalised scaffold. 1,8 bismaleimido diethyleneglycol (BM(PEG)_2_) also enables the functionalisation of thiol-ene polyHIPEs. The functionalisation with BM(PEG)_2_ increases the Young’s modulus by more than three times, however, this results in the scaffold having irreversible deformation when force is applied to it, whereas the unfunctionalised scaffold is able to recover completely after compression and retains its original dimension. BM(PEG)_2_ also affects the cell proliferation in adult CD34^+^ haematopoietic stem and progenitor cells (HSPCs) as it increases significantly when compared to the control scaffolds [78]. Lastly, these types of thiol ene polyHIPEs can also be modified with HA. Lee et al. [79] prepared HA and strontium-modified hydroxyapatite (SrHA) polyHIPE scaffolds from TMPTMP and DPEHA. The scaffolds had a porosity of 90% and a cavity diameter of 58 µm for the non-modified polyHIPE, 57 µm for the 5% HA polyHIPE and 99 µm for the 10% HA polyHIPE. Besides the changes of morphology, the mechanical properties (compressive strength) were also affected. The increase of HA from 5% to 10% increases the compressive strength from 150 to 216 kPa. These scaffolds were also shown to enable the cell adherence and growth of MG63 cells. Due to their porous structure the cells successfully migrated throughout the scaffold. Additionally, cell proliferation was significantly higher on the modified scaffolds when compared to the control scaffold (unmodified).

Besides chemical functionalisation, these scaffolds can also be coated with different molecules. Eissa et al. [80] coated a polyHIPE consisting of TMPTMP and DPEHA with an internal phase volume of 80% and a cavity diameter of approximately 25 µm with fibronectin. The coated scaffolds were shown to have an increased amount of cell adherence of human endometrial epithelial cells (HEECs) and primary human endometrial stromal cells (HESCs) when compared to the uncoated ones. Besides the successful adherence, the cells remained healthy during the entire culture period of 15 d. However, migration into the scaffold was moderate. Nevertheless, these scaffolds are still significantly better than 2-D scaffolds, as the cell morphology in the polyHIPEs is representative of that found in vivo. Lastly, laminin is another molecule that can be used to coat thiol-ene-based polyHIPEs and increase their biological properties. An example of this was done by culturing human induced pluripotent stem cell- and embryonic stem cell-derived neural precursor cells (hPSC-NPCs) on a laminin coated scaffold. The cells were shown to attach, proliferate and differentiate throughout the interconnected structure of the coated polyHIPE [81].

Despite the degradable properties of the thiol-ene polyHIPEs, their degradability is often slow. Thiol-enes based on PETMP and DVA had a mass loss of 45% (6 weeks) in PBS and fully degraded (4 weeks) in 0.01 M NaOH, whereas polyHIPEs based on TMPTMP and TMPTA or DPEHA or HDDA, had a mass loss of 20% (DPEHA) and 30% (TMPTA) in 0.1 M NaOH (7 weeks) and a mass loss of only 1.7% (HDDA) and 3.8% (TMPTA) in PBS after 11 weeks [73,74,75]. Given these properties, Johnson et al. [82] prepared a thiol-ene by incorporating the biodegradable PCL into its structure. This scaffold was shown to readily hydrolyse in NaOH (aq) while producing non-cytotoxic compounds during this process up to a concentration of 0.1 mg/mL. Thiol-ene-based scaffolds used for cell culturing and tissue engineering are summarised in Table 3.

### 2.4. Polyester-Based PolyHIPEs

As already mentioned, biodegradability is a crucial aspect to enable the use of polymers in tissue engineering. However, another integral property is that the products generated during biodegradation are not harmful to the human body. Therefore, the use of FDA approved polymers is highly desirable as they are inherently non-dangerous to human bodies. Examples of such polymers are poly(ε-caprolactone) (PCL), poly (lactic acid) (PLA), polyvinyl alcohol (PVA) and polyacrylamide [86,87]. PCL was used by Lumelsky et al. [88] to prepare PCL-based polyHIPEs with different crosslinkers (tBA, EHA). The tBA polyHIPE had very large pores with a diameter between 1 and 3 mm. Both polyHIPEs were shown to undergo degradation in NaOH. Cell growth experiments were also shown to be favourable on both scaffolds, as the mouse skeletal C2 cells successfully attached to the polyHIPE and formed a monolayer with visible differentiation and myotube formation after a certain time. Additionally, the cells penetrated into the inner parts of both polyHIPEs. Despite similar results, the tBA scaffold was shown to slightly outperform the EHA scaffold. Another PCL based polyHIPE, namely, poly (ε-caprolactone urethane) (PCLU) was prepared from triol PCL oligomers and hexamethylene diisocyanate (HMDI) [89]. Just like the PCL based scaffolds have a non-cytotoxic nature, so do PCLU scaffolds, therefore, using them for tissue engineering applications is favourable [90]. The prepared scaffold had a porosity of approximately 86% and relatively big pore sizes ranging from 150 to 1800 μm. The scaffold was shown to enable cell culture for a period of 7 d with the cell density remaining at approximately 82% compared to the original value [89]. Another way to prepare PCL-based polyHIPEs is by functionalising PCL with methacrylate to prepare a crosslinkable material which undergoes photo crosslinking. Dikici et al. [91] investigated the effect of the solvent ratios on the synthesis of the polyHIPEs. The study demonstrated that the morphology of the polyHIPE is affected by the solvent mixture. An increase of the chloroform ratio increases the average pore and interconnect diameter from 15 and 3.7 μm (60% chloroform, 40% toluene) to 69 and 10.5 μm (100% chloroform), respectively. Consequently, this also affected the elongation which decreased from 84% (100% chloroform) to 66% (60% chloroform). These scaffolds were also shown to enable cell attachment, growth and the infiltration of human dermal fibroblasts (HDFs) comparable to the commercially available Alvetex. Interestingly, the cell distribution throughout the scaffold was more evenly distributed on the smaller pore size scaffolds. The depth of penetration was up to 250 μm for all tested PCL-based scaffolds.

Despite the clear advantages of these biodegradable materials their properties can still be enhanced. Hu et al. [92] have done that by incorporating several different molecules into the scaffold, namely, alginate, bovine serum albumin (BSA) and HA. Additionally, the PCL oligomer had not been chemically modified unlike in the previous examples. The obtained polyHIPE hydrogels were observed to have decreased porosities with the increase of PCL content (91.3% without PCL, 80.8% with 4.5 wt. % PCL). Besides the effect the PCL had on the porosity, it also affected the compressive strength and Young’s modulus of the polyHIPEs which increased with increasing PCL content. The hydrogels had high water uptakes of nearly 1000% after reaching equilibrium. As expected, the PCL content decreased the water up take (lower porosity) which was 600% for 1.5 wt. % and 400% for 4.5 wt. %. Drug release is also crucial to enable the use of artificial scaffolds in tissue engineering, therefore, they were loaded with ibuprofen (IBU). The IBU release showed that the scaffolds had an initial burst release in the first 16 h and a sustained release afterwards. Lastly, mouse bone marrow mesenchymal stem cells (mBMSCs) were cultured on the scaffolds. The cells successfully adhered to the scaffolds, however, increasing the PCL content decreased the cell attachment (hydrophobic character of PCL). Nevertheless, the PCL scaffolds increase the proliferation rate and are viable for tissue engineering. Another way to modify PCL scaffolds using HA is by grafting poly (L lactic acid) (PLLA) on HA nanoparticles (g-HA). To obtain a porous PCL-based scaffold, solvent evaporation was used. The obtained scaffolds had an open porous structure with pore sizes ranging from several microns to over one hundred microns. Additionally, both the increase of PCL content and g-HA nanoparticles decreased the pore size. However, the increase of the internal phase volume increased the pore size. All these parameters enable the synthesis of scaffolds with different pore sizes. g-HA also influenced the Young’s modulus, compressive stress strain (increases with g-HA content) and the biomineralisation activity which is enhanced. Consequently, this could enhance the osteoinductive properties of the scaffold. The IBU release in these scaffolds was shown to have an initial burst release (59.3% of IBU released in 24 h), followed by a sustained release (77.1% IBU released in 240 h). mBMSCs were shown to successfully adhere and proliferate on the scaffold. Increased g-HA content positively affected cell proliferation. The cells were observed to form a spindle-like elongated morphology with lamellipodium at 4 d and formed a confluent layer on the surface at 7 d [93]. Besides PCL, this same method can also be used to prepare scaffolds based on poly (lactic-co-glycolic acid). Similarly, the g-HA nanoparticles increased the Young’s modulus (from 2.4 to 19.2 MPa) and compressive strength (from 0.20 to 1.48 MPa) [94]. This compressive strength is slightly below the compressive strength of cancellous bone, which is between 1.5 and 45 MPa [95]. This scaffold was also tested for drug release and it was shown to have the same type of release as the previous scaffold with an initial burst release (55.3% IBU in 48 h) followed by a sustained release (64.9% IBU in 192 h). This type of release most likely happens due to the release of IBU from the surface of the material which is followed by a slower release due to the increase of the diffusion path length. mBMSCs were shown to form a confluent layer after 7 d, which is similar to the previous scaffold [94]. Another biodegradable polyHIPE can be synthesised from PVA. Unlike all the previously mentioned emulsions, this emulsion system used a gas, namely, CO_2_ as its internal phase to form CO_2_-in-water (C/W) emulsions. By decreasing the PVA content in the aqueous phase the pore size increases from 10 to 20 μm. The scaffolds were shown to enable the proliferation of both human embryonic lung diploid fibroblasts and H9c2 cardiac muscle cells. The number of H9c2 cells increased by 233% after 48 h and by 318% after 72 h [96].

All the mentioned biodegradable materials have been shown to enable the growth of both human and animal cells. Additionally, they had favourable drug release properties and mechanical properties which further increases their applicative possibilities in tissue engineering. Their main advantage when compared to other materials is their inherent biodegradability which gives non-cytotoxic by-products during the degrading process. A summary of polyHIPEs based on polyesters is given in Table 4.

### 2.5. Polysaccharide-Based PolyHIPEs

Most of the previously mentioned scaffolds have been synthesised from synthetic monomers. Despite their degradability, appropriate mechanical properties and their ability to support cell growth, there are still certain limitations of such materials. One of the more integral is their cost which can be relatively high. In comparison, polysaccharides do not have these limitations, as they are abundant in nature. Additionally, polysaccharides are found in living organisms like algae, plants, animals and bacteria [102]. Lastly, they are one of the constituents making up the extracellular matrix (ECM), which makes them highly advantageous for the use in biological applications [103]. PolyHIPEs based on biopolymers/polysaccharides have been synthesised from constituents like gelatine, hyaluronic acid, dextran, pullulan and alginate [104,105,106].

One way to use polysaccharides is as stabilisers in the production of polyHIPEs. By doing this the use of surfactants is usually not needed which is favourable, as surfactants might possess cytotoxic properties. Tan et al. [107] used gelatine nanoparticles to stabilise emulsions containing acrylamide (AAm) in the continuous phase and hexane in the internal phase. The prepared polyHIPE had a cavity diameter of approximately 104 μm. This scaffold was shown to enable the growth of HepG2 cells. Gelatine nanoparticles can also be used to costabilise HIPEs to form gelatine-based polyHIPEs by crosslinking them with genipin. The scaffolds prepared by using this method had an average pore diameter of approximately 25 μm and interconnecting pores ranging from 5 to 10 μm. As the replacement of the scaffold by the seeded cells during tissue engineering is important, the degradation of the scaffold was done with proteinase. It was shown that the higher the gelatine nanoparticle content, the lower the degradation ratio. Lastly, L929 cells were successfully grown on the prepared scaffolds [108]. In addition to gelatine, cellulose can also be used to stabilise Pickering emulsions. Consequently, Liu et al. [109] used supramolecular cellulose nanocrystals (CNCs) which were grafted with a quadruple hydrogen bonding moiety 2-ureido-4(1H)-pyrimidone (UPy) to obtain UPy modified CNCs (CNC-UPy). These nanoparticles were used to stabilise a polyHIPE consisting of AAm, gelatine and N,N′-methylenebisacrylamide (MBAA). To enable a successful polymerisation, the gelatine was modified with methacrylic anhydride to prepare gelatine methacrylate (GelMA). The resulting hydrogels had a decreased average pore size with increased CNC-UPy content (decreased from approximately 60 to 30 µm with an increase of CNC-UPy from 0.1 to 0.5 wt. %). These hydrogels were also able to absorb large amounts of water in a short time period (almost 40 g/g in the case of 0.1 wt. % CNC-UPy). The polyHIPEs enabled a significantly higher growth of mBMSCs after 5 d when compared to the non-porous hydrogel. This is another confirmation that 3-D polysaccharide-based scaffolds are also better than 2-D scaffolds in cell growth applications. Gelatine can also be combined with other components, for example, hyaluronic acid (Ha) and chondroitin sulfate (CS) to prepare porous scaffolds. The obtained scaffold had a pore size of 28 µm and an interconnect size of 17.5 µm with a nominal porosity of 90%. The cells successfully attached to the scaffold and were shown to have good viabilities. The observations showed that the cells formed numerous microvilli which is distinct for healthy cells. Additionally, albumin production was also increased [104]. These scaffolds were further researched in a different study by Colli et al. [110], where they increased the pore diameter from a range of 10 to 20 μm to a range of 10 to 50 μm with the addition of dimethyl sulfoxide (DMSO) and NaCl to destabilise the emulsion. This scaffold was shown to have a good initial adhesion and maintained a good viability for the primary rat hepatocytes and the hepatic cell line, C3A, during the testing period. Both cell types were shown to have better cell viability on the prepared 3-D scaffolds than the monolayer polystyrene plates. The main drawback of this method to increase the pore size is the use of an organic solvent which might affect the biocompatibility. Besides gelatine, there are also other compounds which can be used to prepare porous scaffolds for biological application. Another example of such scaffolds are dextran and pullulan which were functionalised with vinylic functionalities. The prepared scaffolds had favourable properties for the growth of a primary cell culture (mixed glial cells and neurons). The neurons were able to colonise the inner cavities, whereas the glial cells adhered to the surface of the scaffold, as they are larger (40–60 μm) than the average cavity diameter (20 μm) [105].

**Figure 3:**
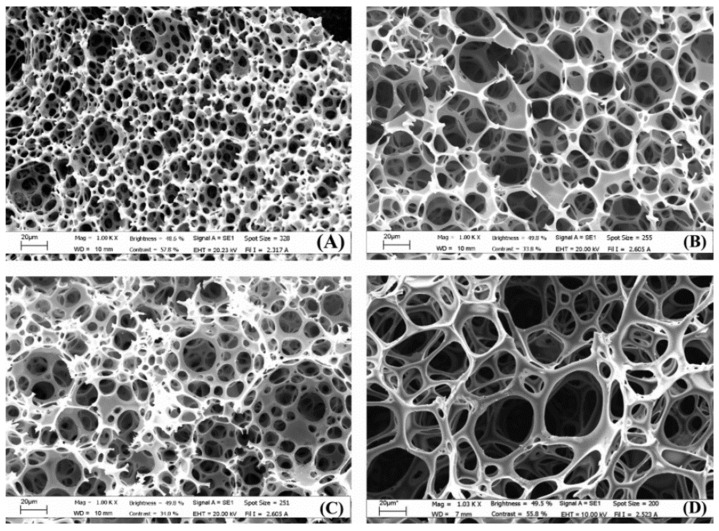
SEM images of polyHIPEs prepared with radical polymerisation and different nominal porosities: (**A**,**C**) 90%; (**B**,**D**) 92%. Samples (**C**,**D**) were prepared with 0.01 M NaCl and 1% *v*/*v* DMSO. Used with permission from reference [111].

Polysaccharide-based scaffolds can be prepared by different crosslinking methods, namely, free radical polymerisation, aldol condensation and even enzymatic crosslinking. Barbetta et al. [111] evaluated radical polymerisation and enzymatic cross-linking (Figure 3). Both materials were found to be viable for the culture of primary rat hepatocytes. However, the enzymatic polyHIPEs had a higher cell loading efficiency and a higher cell viability at 96 h. Additionally, the MTGase scaffolds had better phenotype expression than the scaffolds prepared by radical polymerisation. This is most likely due to the absence of any foreign chemical functionalities, enabling the use of the biocompatible nature of gelatine to its fullest. A summary of polysaccharide-based polyHIPEs is shown in Table 5.

## 3. Combined and Advanced Applications

HIPE templating can be combined with more advanced manufacturing methodologies, for example, electrospinning, hard sphere templating and additive manufacturing. Combining these methods enables the production of more advanced materials.

HIPE templating can be combined with electrospinning to form a bilayer. Such a bilayer would enable cell growth in the polyHIPE and prevent penetration into the electrospun layer, which would act as a physical barrier that allows nutrient diffusion. An example of such a scaffold was prepared by Dikici et al. [97]. The polyHIPE and electrospun layer were both based on PCL. The polyHIPE layer was shown to enable the growth of MLO-A5 cells with a gradual increase in metabolic activity from day 1 to day 28. Additionally, the plasma treated scaffold enabled a cell penetration of up to a depth of 400 µm after 4 weeks, whereas the electrospun layer prevented cell penetration while allowing nutrient diffusion. This same method was used to prepare another bilayer polymer. The polyHIPE layer was made up of PCL while the electrospun layer consisted of poly3 hydroxybutyrate-co-3-hydroxyvalerate. This scaffold was shown to promote proliferation and migration of human aortic endothelial cells (HAECs) [98].

**Figure 4:**
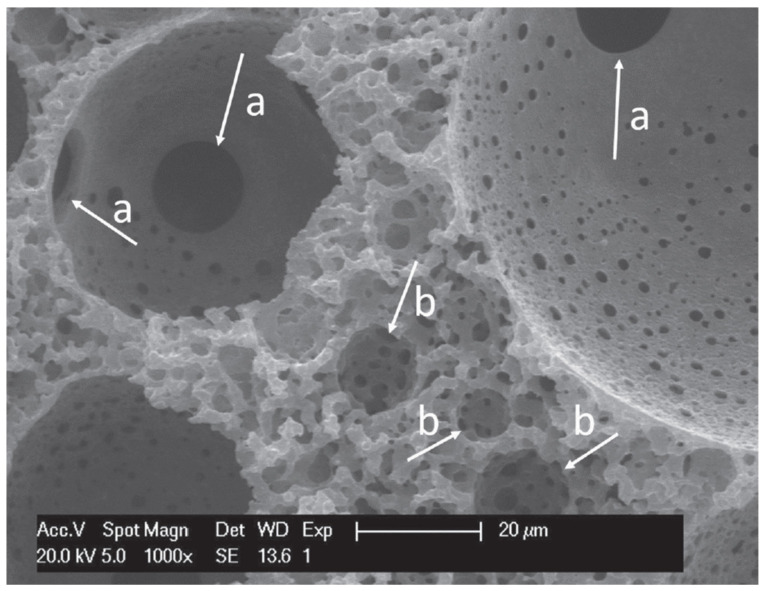
SEM image of: (a) interconnecting pores of the primary pores (hard sphere templating); (b) secondary pores (HIPE). Used with permission from reference [83].

A higher level of macroporosity can be introduced into polyHIPEs by combining hard sphere templating with emulsion templating. This is done by preparing a 3-D monolithic structure consisting of polymer spheres based on poly (methyl methacrylate) (PMMA) through sintering [113]. This structure was used as a scaffold through which a HIPE based on PETMP and DVA was poured. After the polymerisation the PMMA beads were removed and a multilevel hierarchical porosity was obtained. This material had a primary pore size of 70 µm (PMMA beads) and a secondary pore size of 7 µm (HIPE droplets) which were connected with interconnecting pores (Figure 4). As such a material mimics many of the naturally available biomaterials it was evaluated for possible tissue engineering applications by using human bone derived cells (osteoblasts). The material enabled cell growth on both the surface and the inner parts of the scaffold [83].

**Figure 5:**
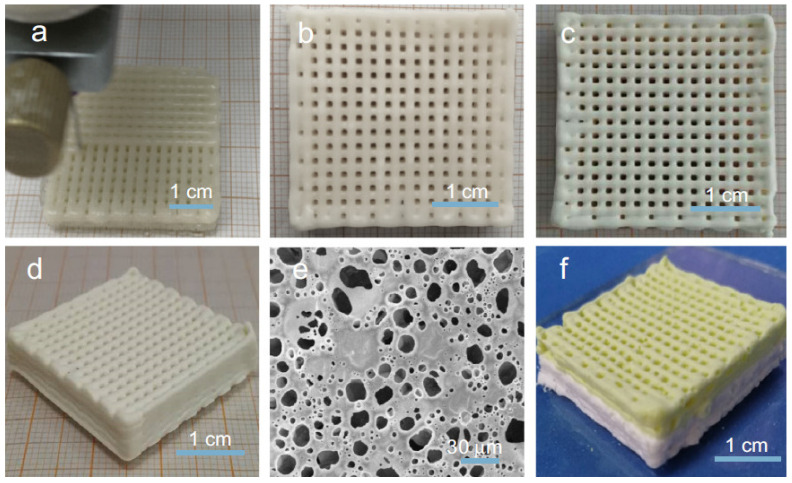
(**a**) printing of the scaffold; (**b**–**d**) photos of the dried scaffold; (**e**) SEM image of the printed scaffold; (**f**) photo of the composite scaffold (constructed from different precursor HIPEs). Used with permission from reference [99].

Besides PMMA beads, alginate beads can also be used to incorporate a multilevel porosity into the scaffold. These beads were added to the HIPE and after the polymerisation they were removed. The resulting scaffold has a multilevel porosity with pore sizes of up to 780 µm (alginate beads); in the range of 10 to 50 µm (HIPE droplets) and in the range of 1 to 10 µm (interconnecting pores). This scaffold enhanced initial cell seeding efficiency, promoted cell ingrowth (up to a depth of 450 µm) and enabled uniform matrix deposition [59]. Another method that facilitates the formation of a multilevel porosity is 3-D printing. Owen et al. [61] used stereolithography to polymerise polyHIPEs based on EHA and IBOA which resulted in the formation of a multilevel porous scaffold. The primary level of porosity was due to the fabrication of a woodpile scaffold (300 μm vertical; 650 μm lateral), the secondary with a size ranging from 20 to 30 μm was due to the HIPE droplets and the tertiary level was due to the interconnecting pores. This study also showed that there was no significant difference in the metabolic activity oh hES-MPs between the air plasma treatment and plasma deposited acrylic acid treatment. Another polyHIPE that can be obtained with stereolithography is based on IBOA and TMPTA. A woodpile structure was produced to form a multilevel porous scaffold. This scaffold was compared to a commercially available scaffold (3D Insert). The polyHIPE based material outperformed the commercially available scaffold, as the metabolic activity and mineralisation of MLO-A5s were higher [65]. These types of scaffolds were also shown to enable the growth of MG63 cells [66]. Besides stereolithography, 3-D printing can also be conducted through extruding, however, the emulsion needs to have an appropriate viscosity to enable the successful utilisation of this method. Hu et al. [99] prepared a 3-D printed PCL based polyHIPE which was modified with HA (Figure 5). As one of the intended usages of this scaffold is as an implant, it was loaded with IBU to reduce inflammation. The scaffold was shown to successfully release IBU in a controlled manner (initial burst release followed by a sustained release). Lastly, the scaffold enabled the mBMSCs to spread uniformly and the formation of a spindle shape. Dikici et al. [100] also used a PCL-based HIPE for 3-D printing applications. Besides the printing of a woodpile scaffold (multilevel porosity) an ECM was generated by using MLO-A5 cells. The cells were firstly cultured on the scaffold and then decellularised to form a biohybrid scaffold. This scaffold was then seeded with hES-MPs and compared to a PCL-based scaffold. This biohybrid material was shown to have three-times higher cell attachment and significantly higher cell viability. Additionally, the biohybrid scaffold had good angiogenic performance, as the amount of blood vessels, total vessel length and the total number of junctions in comparison to the control group increased significantly. Yang et al. combined PCL and PLLA to produce a 3-D printed scaffold. This scaffold was shown to efficiently release enrofloxacin (ENR) (80% release in 2.5 h; 98% release in 10 h) and to enable the adherence and proliferation of mBMSCs [101]. Whitely et al. [84] also prepared a 3-D printed polyHIPE scaffold. However, they combined a polyHIPE based on PFDMA with a hydrogel carrier which was used to deliver human mesenchymal stem cells (hMSCs) in situ. The hydrogel successfully released hMSCs uniformly onto the 3-D printed polyHIPE without affecting the morphology. In comparison, suspension seeding caused an irregular cell distribution and lower cell densities.

**Figure 6:**
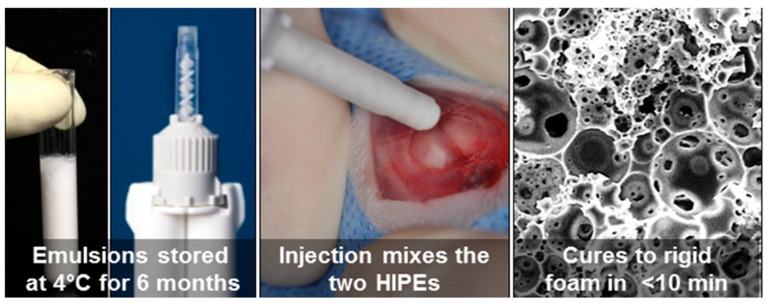
Injectable double barrel system to enable long storage and fast curing upon injection [57]. (Used with permission from ACS Publications. Further permissions related to the material excerpted should be directed to the ACS).

Given that certain HIPEs have appropriate viscosities to be used in 3-D printing, they can also be used as injectables as they can fill irregular defects after being injected. Additionally, they can be polymerised in vivo. Cosgriff-Hernandez’ group prepared injectable polyHIPEs based on PFDMA which polymerised at 37 °C and produced a closed-porous material with pores ranging from 4 to 29 μm with a compressive strength of 5 MPa (in the range of cancellous bone). The scaffold was shown to have a high cell viability (95%) for 3T3 fibroblasts [67]. To obtain an open porous polyHIPE based on PFDMA, an organic-phase soluble initiator was used (benzoyl peroxide (BPO)), unlike in the previous case, where a water-soluble initiator was used. This scaffold also had mechanical properties comparable to cancellous bone. Interestingly, this HIPE could be stored for a period of 1 week at 4 °C without significantly affecting the pore morphology. This enables the use of this material as an off-the-shelf bone graft. The HIPE integrated well with native tissues in a porcine femur defect. It was able to resist flow, even after being inverted, which indicated that the slow cure time of PFDMA HIPEs might not be an issue, as the material remained in the defect during the curing process. The scaffold had a high hMSC cell viability of 90% [68]. The PFDMA-based scaffold was further improved by using redox-initiated polymerisation to shorten cure times. This was conducted by using a double-barrel syringe system. Each of the barrels contained the macromer PFDMA and different initiators (BPO in one barrel and trimethylaniline (TMA) in the other barrel). Upon injection, the HIPEs are mixed and polymerisable at 37 °C. The main advantage of this system is the ability to store the unpolymerised HIPEs for a period of 6 months with minimal effects on the properties and the ability to solidify in less than 15 min (Figure 6). The mechanical properties of this material also matched those of cancellous bone. As this system is meant to be used as an injectable bone graft, it was crucial to study the cell viability of hMSCs on the unreacted macromers. PFDMA was shown to have a cell viability of above 80%, whereas the other monomers had lower viabilities (BDMA and EGDMA). However, diluting the monomers results in cell viabilities above 80% which is considered to be cytocompatible [57]. Despite the good properties of the PFDMA-based polyHIPEs, they still possess a slight disadvantage, namely, the susceptibility of the redox-initiated polymerisation to oxygen inhibition. To prevent oxygen inhibition, thiol-based scaffolds can be used, as they have increased resistance to oxygen inhibition. PFDMA was combined with PETMP to form a HIPE with an internal phase of 75%. Some other advantages of thiol-ene polyHIPEs are their rapid polymerisation rates and their tunable degradation profile, resulting in the full degradation of the polyHIPE in 0.5 M NaOH after 3 weeks. Lastly, this scaffold was viable for hMSC cells [85]. Additionally, PFDMA-based injectables can be improved by incorporating particles (calcium phosphate nanoparticles and demineralised bone matrix) into the structure. The particles have negligible effect on the pore morphology and compressive properties, while forming scaffolds with high cell viabilities (hMSCs) and increased cell proliferation rates due to the presence of osteoinductive particles [63]. Injectable HIPEs can also be prepared from polysaccharides. Zhou et al. [106] used an alginate-based HIPE to form hydrogels. These hydrogels had a water uptake of approximately 8000% (*w*/*w*) and were successfully colonised by A549 cells with a 300% higher cell proliferation than the positive control material. As the main use of this material was in the form of an injectable it was loaded into a syringe and successfully extruded. However, after the extrusion, the hydrogel particles broke into smaller fragments. Nevertheless, it was able to fill the confined space of pork muscle. To enable the formation of a single monolithic particle, a solution of alginate was used as an adhesive to bind the hydrogel fragments in the presence of Ca^2+^. The hydrogel was held together by the alginate without significantly affecting the morphology or cell growth. A drawback of using HIPEs as injectable is the possible cytotoxicity of the surfactants. Due to that, Oh et al. [112] stabilised the HIPEs with gelatine graft poly(N-isopropylacrylamide) (GN). These polyHIPEs were able to proliferate human foreskin fibroblast cells. Lastly, injectables can be improved by incorporating growth factors into them, for example, bone morphogenetic protein 2 (BMP-2). Whitely et al. [56] have done that by loading polyHIPE microspheres with BMP-2 and incorporating them into a scaffold. BMP-2 is released in a controlled manner into the scaffold and promotes the osteoblastic response of hMSC cells. The microspheres had no significant effect on the compressive properties of the polyHIPE. The main advantage of combining two different systems is the ability to control the properties of each system separately.

Given that polyHIPEs are inherently porous, an application where these properties could be excellently applied is wound dressing. Materials used in wound dressing are required to have absorptive and haemostatic properties. McGann et al. [60] prepared a PEGDA-based polyHIPE. This polyHIPE had buffer uptakes of up to 65.5 g/g and 38.9 g/g in the case of the addition of PNIPAM. The scaffolds were shown to be cytocompatible. Additionally, the cells did not spread in great numbers on the surface of the polyHIPE, however, this is suitable for wound dressing materials, as cell adherence might complicate later dressing removal. Another property that is required of these materials is good blood-clotting efficiency. The acrylate-only and PNIPAM-containing scaffolds were compared to rayon gauze and significantly outperformed the commercially available product. Rayon gauze had a blood-clotting index of 36.7%, whereas acrylate-only and PNIPAM-containing materials had an index of 81.3% and 93.4%, respectively. As the presence of antibiotics in wound dressing materials is favourable (slows down the infection), the scaffolds were loaded with ciprofloxacin or tetracycline. Both scaffolds inhibited the growth of *Staphylococcus aureus*, while having a characteristic burst-release of the antibiotics. Another material that could potentially be used for wound dressing application is polyacrylate (consisting of different monomers: BA, TMPTA, GMA, HDDA, PEGMA). Based on that, the drug delivery abilities of the scaffold were evaluated by using a bioactive molecule (curcumin). The molecule was released in a controlled way. Firstly, there was a burst release which was followed by a constant release rate until the full release of the bioactive molecule (100–140 h). The scaffold was also shown to be cytocompatible with human fibroblasts. The last study conducted was done on the angiogenic properties of the material which were neither pro- nor anti-angiogenic. Due to the demonstrated properties, this material would be suitable for wound dressing applications [69]. The last material found in the literature that was tested for wound dressing applications is based on both PEGMA and PEGDA. This scaffold was loaded with 0 wt. %, 50 wt. % and 100 wt. % kaolin. Kaolin did not affect the pore size and had negligible effect on the absorption rates (favourable absorption rates when compared to commercially available gauze and foam dressing materials). As non-cytotoxicity is mandatory, it was also evaluated using HDFs and showed that the material is not cytotoxic. Additionally, the scaffold was not adherent for HDFs which is, as we have already mentioned, beneficial for wound dressing applications. The 100% kaolin loaded polyHIPE had a 41% reduction of blood clotting which is comparable to commercial wound dressing materials [64].

Lastly, polyHIPEs were used in the form of a microfluidic-based tissue-on-a-chip device. Claeyssens’ group prepared such a scaffold based on EHA, IBOA and TMPTA. This polyHIPE can be used as an osteogenesis-on-a-chip device for long-term cell culture, which can potentially replace animal testings, as it can mimic the 3-D environment present in tissues and organs [70].

## 4. Conclusions

An increasing number of reports on using porous polymers from high internal phase emulsions in biomedical applications, particularly as scaffolds for biological cell growth and tissue (re)generation, show the importance and suitability of this type of macro porous polymer in the biomedical field. Lately, techniques which combine the use of high internal phase emulsion templating with another material structurisation methods have been developed to facilitate the synthesis of tailor-made polymer materials. The cellular open porous structure of polyHIPEs and polymers prepared via combined approaches, the possibility of tuning the pore size distribution and pore volume, the possibility of use of biocompatible and biodegradable chemistries and the adaptation of mechanical properties, make polyHIPEs and polyHIPE-derived materials a very attractive choice as materials for biomedical applications.

## Figures and Tables

**Table 1:** Styrene-based polyHIPEs used in cell culturing and tissue engineering. Samples marked with * have had their porosity measured, whereas the non-marked ones are based on the nominal porosity.

Monomers	Porosity (%)	Pore Size (μm)	Cells	Reference
STY; DVB; EHA	90	N/A	HepG2	[37]
STY; DVB	95	N/A	HepG2	[38]
STY; DVB; EHA	90	≈90	MG63	[39]
STY; DVB	95 *	≈12	A549	[40]
STY; EGDMA	90	N/A	L929	[41]
STY; DVB; EHA	89–92 *	≈19; ≈60	Hepatocytes	[42]
STY; DVB; EHA; PFPA	90	≈69	HepG2	[43]
STY; DVB	95	≈100; ≈60; ≈40	Primary rat osteoblasts	[45]
STY; EGDMA	90	30–40	L929	[46]
PS-PAA; PS-PEO	80	40–120	hES-MP	[47]
PS-PAA; PBD-PEO	90	40–80	hES-MP	[48]
STY	95	≈100; ≈40	Rat osteoblasts	[49]
STY; DVB	90	5–20	TERA2.cl.SP12	[50]
STY; DVB	90	5–20	TERA2.cl.SP12	[51]
STY; PCL/PLA	90	N/A	Several	[52]
STY; PCL; MMA	75–90	5–100	fibroblasts	[53]
STY; DVB	95	≈100	Primary rat osteoblasts	[54]
STY; PCL	88	18–30	Mouse skeletal cells (C2)	[55]

**Table 2:** Acrylate-based polyHIPEs used in cell culturing and tissue engineering. Samples marked with * have had their porosity measured, whereas the non-marked ones are based on the nominal porosity.

Monomers	Porosity (%)	Pore Size (μm)	Cells	Reference
EGDMA	75	≈12, ≈14, ≈29	hMSC	[56]
PFDMA; EGDMA; BDMA	75	≈5	hMSC	[57]
GMMA; GDMA; HEMA	92–97 *	20–30	NIH/3T3	[58]
EHA; TMPTA	90	≈780; 10–50	MLO-A5	[59]
PEGDA; Na/Ca Acrylate; PNIPAM	N/A	32–44	HeLa cells	[60]
EHA; TMPTA; IBOA	75–90	20–30	hES-MP	[61]
EHA; IBOA; TMPTA	80	≈25	hES-MP	[62]
PFDMA; BDMA	75	7–9	hMSC	[63]
PEGMA; PEGDA; Na Acrylate	83	≈3	HDFs	[64]
IBOA; TMPTA	80	≈40	MLO-A5	[65]
EHA; IBOA; TMPTA	80	≈33	MG63	[66]
PFDMA	75	4–29	NIH/3T3	[67]
PFDMA	75	≈12	hMSC	[68]
GMA; BA; TMPTA; HDDA; PEGMA	80	N/A	Human fibroblasts	[69]
EHA; IBOA; TMPTA	80	≈16	hES-MP	[70]

**Table 3:** Thiol-ene-based polyHIPEs used in cell culturing and tissue engineering.

Monomers	Porosity (%)	Pore Size (μm)	Cells	Reference
PETMP; DVA	85	≈13	MC3T3-E1	[72]
PETMP; DVA	85	≈82	Chondrocyte	[73]
TMPTMP; TMPTA; DPEHA	90	≈108	HaCaTs	[74]
TMPTMP; TMPTA; HDDA; PEGDA	80/85	≈30; ≈44; ≈45; ≈63	iPSC-hNPCs	[75]
TMPTMP; TMPTA	80	20–30	HESCs	[76]
TMPTMP; TMPTA	80	≈38	WA09 (H9)	[77]
TMPTMP; DPEHA	90	≈37	(CD34^+^) HSPC	[78]
TMPTMP; DPEHA	90	≈58, ≈57, ≈99	MG63	[79]
TMPTMP; DPEHA	80	≈25	HEECs, HESCs	[80]
TMPTMP; PEGDA; TMPTA	80	N/A	hPSC-NPCs	[81]
TMPTMP; PCL; TMPTA; DPEHA	90/95	≈60	L929	[82]
PETMP; DVA	90	≈70	Osteoblasts	[83]
DTT; PEGDA	75	N/A	hMSCs	[84]
PETMP; PFDMA	75	≈6	hMSCs	[85]

**Table 4:** Polyester-based polyHIPEs used in cell culturing and tissue engineering. Samples marked with * have had their porosity measured, whereas the non-marked ones are based on the nominal porosity.

Monomers	Porosity (%)	Pore Size (μm)	Cells	Reference
AAm	≈90 *	≈10; ≈17; ≈19	H9c2	[87]
PCL; tBA; EHA	88	1000–3000	Mouse skeletal cells (C2)	[88]
PCL; HMDI	75	150–1800	hMSCs	[89]
PCL	82	≈15; ≈20; ≈69	HDFs	[91]
PCL	81–91 *	Tens of micrometres	mBMSCs	[92]
PCL	90 *	Few μm to a few 100 μm	mBMSCs	[93]
PLLA; PLGA	86 *	N/A	mBMSCs	[94]
PVA; Glutaraldehyde	80	≈10; ≈20	Fibroblasts/H9c2	[96]
PCL	85	≈34	MLO-A5	[97]
PCL	80	≈30	HAECs	[98]
PCL; PLLA	96*	N/A	mBMSCs	[99]
PCL	89	≈8	MLO-A5	[100]
PCL; PLLA	75	10–30	mBMSCs	[101]

**Table 5:** Polysaccharide-based polyHIPEs used in cell culturing and tissue engineering.

Monomers	Porosity (%)	Pore Size (μm)	Cells	Reference
Gelatine; HA; CS	90	≈28	C3A/HepG2	[104]
Dextran; Pullulan	90/95	≈20	Mouse neural cells	[105]
Alginate	N/A	7–12	A549	[106]
Gelatine; AAm; MBAA	80	≈104	HepG2	[107]
Gelatin	80	≈25	L929	[108]
Gelatine; AAm; MBAA	80	30–62	mBMSCs	[109]
Gelatine; HA; CS	90	10–20; 10–50	C3A	[110]
Gelatine	90/92	≈60; ≈84	Rat hepatocytes/HepG2	[111]
Gelatine; PNIPAM	95/96	≈70; ≈80	Foreskin fibroblasts	[112]

## Data Availability

Not applicable.
